# Observation for the feasibility of a bile duct biopsy technique using a loop-tip guidewire during endoscopic retrograde cholangiopancreatography

**DOI:** 10.1097/MD.0000000000030784

**Published:** 2022-10-14

**Authors:** Kang Won Lee, Jae Min Lee, Hyuk Soon Choi, Eun Sun Kim, Bora Keum, Yoon Tae Jeen, Hoon Jai Chun, Hong Sik Lee

**Affiliations:** a Division of Gastroenterology and Hepatology, Department of Internal Medicine, Korea University Anam Hospital, Seoul, Republic of Korea.

**Keywords:** endoscopic retrograde cholangiopancreatography, forceps biopsy, guidewire assistance

## Abstract

Endoscopists frequently have difficulty approaching biliary lesions using biopsy forceps. The aim of this study was not only to describe an easy technique for biliary biopsy assisted by a looped guidewire but also to present preliminary results regarding its safety and feasibility. A preliminary proof-of-concept study was performed at a single tertiary medical center. Between August 2019 and January 2020, 13 patients with bile duct strictures underwent endoscopic retrograde cholangiopancreatography (ERCP) with a new loop guidewire-assisted forceps approach technique. The efficacy and safety were evaluated using the success rate as the primary outcome and diagnostic yield and complication rates as secondary outcomes. The tissue sampling success rate was 100% (13/13). All samples were acceptable for histopathological analysis. Eleven specimens were confirmed to be adenocarcinomas. After reexamination of the remaining 2 patients, all cases were eventually diagnosed as being malignant. The sensitivity of the single procedure was 84.6% (11/13). There were 2 patients with mild hyperamylasemia, but there were no severe complications with respect to safety. This new technique could enhance the success rate and diagnostic yield and reduce the risk of failure when using the biopsy forceps approach during ERCP.

## 1. Introduction

Endoscopic retrograde cholangiopancreatography (ERCP) has been a very important method for the diagnosis and treatment of bile duct diseases.^[1]^ With the recent development of various accessories, ERCP is playing a more important role.^[2]^ In bile duct stenosis, tissue diagnosis is essential because treatment options, such as surgery, chemotherapy, and radiation therapy, can vary depending on the pathology report results.

However, the procedure for collecting samples during ERCP is difficult because it requires considerable experience. Although brush cytology through ERCP is mainly used in the clinical world, the diagnostic yield is relatively low for identifying the cause of biliary strictures. According to a previous review, the sensitivity of brush cytology during ERCP was 6% to 64%, with a very large variation between studies, and the average was reported to be as low as 41.6% ± 3.2%.^[3]^ In a recent study, the sensitivity of brush cytology was found to be higher, at 74.6%, with the help of rapid on-site evaluation, but it is difficult to generalize these results because the number of institutions equipped for conducting rapid on-site evaluation are limited.^[4]^

Some advantages of the biopsy method include a higher diagnostic rate and negative predictive value compared with those of the brush cytology method.^[5]^ Although forceps biopsy is a commonly performed procedure with these advantages, if the location of the stricture cannot be easily approached with biopsy forceps, endoscopists may find this technique difficult. In particular, novice endoscopists who have just begun learning ERCP may have difficulty inserting biopsy forceps through the papilla, which may cause unnecessary complications such as procedure-related bleeding or mechanical injury to the papilla.^[6]^ In addition, several samples should be retrieved to increase diagnostic rates.^[7]^

The aim of this study was to ensure that bile duct biopsies can be performed easily and safely using the new loop-tip guidewire approach.

## 2. Materials and Methods

### 2.1. Patients

Between August 2019 and January 2020, our refined method of trans-papillary bile duct biopsy was attempted in 13 selected cases with extrahepatic or intrahepatic bile duct strictures detected by ERCP. An ERCP expert selected 32 candidate patients based on cholangiograms after cannulation, whose biliary stricture lesions were considered difficult to access using biopsy forceps with the conventional method. Of the 69 consecutive patients who were seen during the study period, 32 were selected by the ERCP expert based on the status of the papilla and bile duct. Patients with small papillae, intra-diverticular or peri-diverticular papillae, malignant infiltration or distortions of the papillae, or tortuous bile ducts were included as candidates. A novice endoscopist (who had performed < 100 ERCP procedures) was given 5 minutes to try to approach the biliary stricture lesion under the supervision of the ERCP expert. For 13 patients, the conventional method of using forceps to approach the biliary stricture lesions in under 5 minutes failed, and they were enrolled in the study. Thus, of the total 69 patients, 32 candidates were chosen by an ERCP expert, of which 13 underwent the loop-tip guidewire approach.

### 2.2. Study design

We conducted a preliminary study to determine the safety and feasibility of using the loop-tip wire for easy insertion of biopsy forceps. Sensitivity was measured based on the pathological results of initially obtained bile duct tissue, and the incidence of post-ERCP complications was also evaluated. The diagnosis of post-ERCP pancreatitis was based on the consensus definition and classification proposed by Cotton et al, which states the following: new or worse abdominal pain after ERCP, and serum amylase at least 3 times the normal upper limit, resulting in the prolongation of the planned hospitalization period by more than 2 days.^[8]^ This study was approved by the Institutional Review Board, Korea University (IRB number: 2019AN0313).

### 2.3. Procedure

All patients provided written informed consent before undergoing ERCP and biopsy. A conventional duodenoscope with a working channel 3.2 mm in diameter (TJF-260V; Olympus Optical, Tokyo, Japan) was used. A standard catheter for cannulation was inserted into the bile duct. A cholangiogram image was obtained to detect any bile duct stricture, after which a 0.035-inch or 0.025-inch-diameter polymer-coated guidewire (Jagwire™; Boston Scientific, Marlborough) was passed through the stricture lesion. After passage of the guidewire, the ERCP catheter was withdrawn. An endoscopic sphincterotome (Jagtome™ RX; Boston Scientific, Marlborough) was inserted along the pre-installed guidewire. An endoscopic sphincterotomy was performed. A loop-tip wire (Fusion® LoopTip™; Cook Medical, Bloomington) was then prepared to perform a loop guidewire-assisted biopsy.

**Figure 1.**
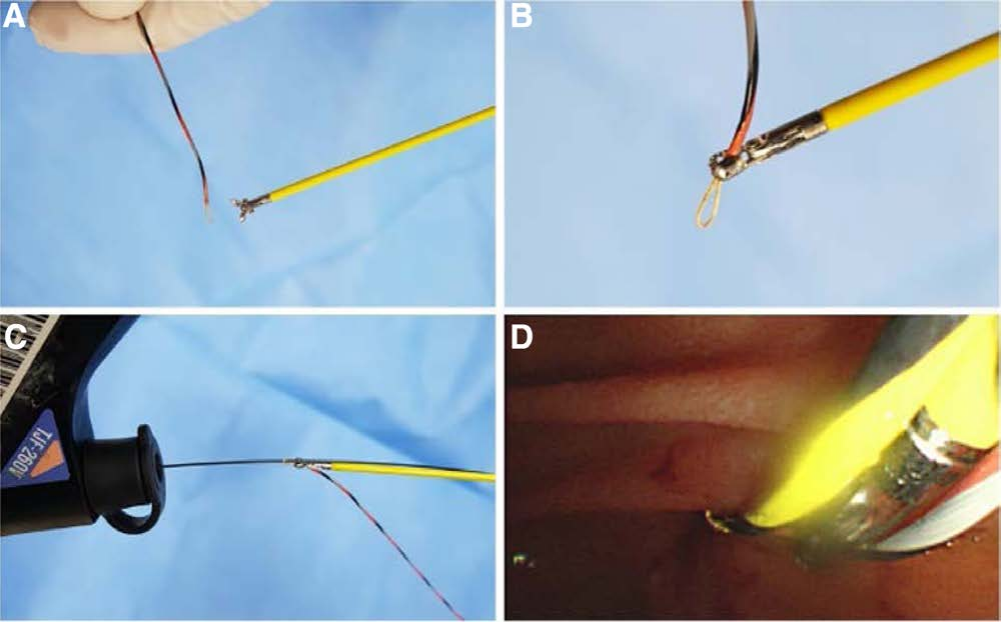
Preparation for the loop-tip guidewire-assisted biopsy forceps approach. A loop-tip guidewire is prepared (A). Biopsy forceps are used to grasp the end of the loop-tip guidewire (B). The pre-installed guidewire is inserted into the loop (C). The loop-tip guidewire with the biopsy forceps is advanced into the duodenal lumen, right along the pre-installed guidewire (D).

The process of the new technique is as follows: the biopsy forceps (Radial Jaw™ 4; Boston Scientific, Marlborough) are manipulated to grab the distal end of the loop-tip wire; the pre-installed main guidewire is then inserted into the loop of the loop-tip wire; with the forceps holding the loop-tip wire, it is inserted through the bile duct along the pre-installed guidewire; when the forceps reach the target lesion, the loop-tip wire is released and pulled out; and a bile duct biopsy is performed under fluoroscopic guidance. The forceps insertion procedure described above is shown in Figure 1.

**Figure 2.**
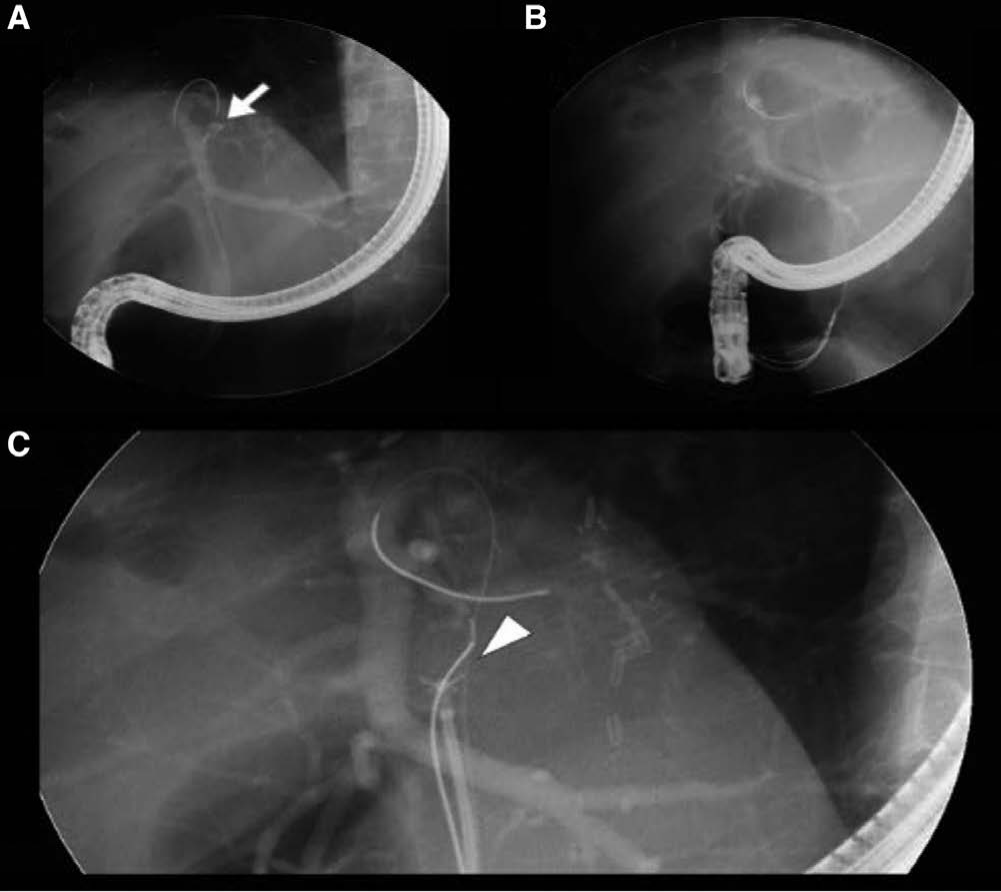
The loop-tip guidewire-assisted biopsy forceps approach process. A pre-installed guidewire is placed across the stricture lesion of the bile duct (A). A loop-tip guidewire with biopsy forceps is advanced along the pre-positioned guidewire until it approaches the stricture lesion (B). The forceps holding the loop-tip guidewire is released from the loop-tip guidewire and pre-installed guidewire, allowing for free manipulation of the biopsy forceps (C).

At least 3 specimens were obtained from each patient to secure an adequate and sufficient amount of tissue. The obtained specimen was transferred to a pathologist, and the “unsatisfactory” specimen, which the pathologist could not perform microscopic analysis, and the “non-diagnostic” specimen that could be analyzed but had insufficient information to make a diagnostic classification were judged as inadequate specimens. During and after the biopsy procedure, the pre-installed main guidewire position remained unchanged. Additionally, the forceps grasping the loop-tip wire were not separated in the channel of the scope by holding the forceps handle. Figure 2 shows a fluoroscopic image of the insertion and release of the biopsy forceps from the loop-tip wire and pre-installed guidewire. At the end of the procedure, plastic stents were introduced into the proximal side of the stricture lesion along the pre-installed guidewire.

## 3. Results

The biliary stricture lesions of all 13 patients were located proximal to the cystic duct insertion site (10 in the common hepatic duct [CHD] and 3 in the intrahepatic duct [IHD]). There were 9 male and 4 female patients, with a mean age of 68.2 ± 12.1 years. Laboratory blood tests revealed that the majority of the enrolled patients had liver function test abnormalities showing an obstructive pattern. The average and standard deviations of laboratory findings and baseline characteristics of the enrolled patients are summarized in Table 1.

**Table 1:** Baseline characteristics of the enrolled patients.

	Value
Patient, *n*	13
Age, yrs	68.2 ± 12.1
Male, *n* (%)	9 (69)
**Location of biliary lesion, *n* (%**)	
Common hepatic duct	10 (77)
Intrahepatic duct	3 (23)
**Laboratory findings**	
WBC, ×10^3^/L	6.31 ± 2.07
AST, IU/L	116.4 ± 80.6
ALT, IU/L	121.2 ± 101.3
ALP, IU/L	404.3 ± 259.7
GGT, IU/L	497.7 ± 296.8
Total bilirubin, mg/dL	7.5 ± 6.7
Direct bilirubin, mg/dL	4.5 ± 4.9
BUN, mg/dL	15.1 ± 4.0
Creatinine, mg/dL	0.8 ± 0.2
Amylase, IU/L	75.9 ± 68.6
Lipase, IU/L	143.2 ± 320.3

ALP = alkaline phosphatase, ALT = alanine aminotransferase, AST = aspartate transaminase, BUN = blood urea nitrogen, GGT = gamma-glutamyl transpeptidase, WBC = white blood cell.

For all 13 patients, the biopsy procedure via ERCP was performed without any specific complications. A sufficient amount of tissue was obtained in all 13 patients, and the pathologist assessed all tissues as adequate specimens for histologic evaluation. The histological diagnosis of cancer was eventually made in 11 of the 13 patients with malignant biliary strictures. The remaining 2 patients showed chronic inflammation with fibrosis on pathology reports. The sensitivity of the test was 84.6% (11/13). However, the remaining 2 patients showed apparent progression of multiple liver metastases or solid masses, suggestive of cholangiocarcinoma on computed tomography. Therefore, we decided to perform an ultrasound-guided liver biopsy and reattempt a bile duct biopsy by ERCP again for proper diagnosis with acquisition of adequate specimens. The final diagnoses were as follows: CHD cholangiocarcinoma (n = 9), pancreatic cancer with liver metastasis (n = 1), gallbladder cancer (n = 1), hepatocellular carcinoma combined with cholangiocarcinoma (n = 1), and recurrent IHD cholangiocarcinoma (n = 1). The time from confirmation that the pre-installed guidewire had passed through the stricture lesion till the biopsy forceps reached the stenotic lesion with the help of the loop-tip guidewire was calculated. The average calculated time was 193.0 ± 82.1 sec, though the absolute insertion time was shorter. The final pathological diagnostic results of the enrolled patients are summarized in Tables 2 and 3.

**Table 2:** Endoscopic biliary biopsy outcomes.

	Value
Success rate, *n* (%)	13 (100)
Post-ERCP complications, *n* (%)	0 (0)
**Pathologic findings via endoscopic biopsy, *n* (%**)	
Adenocarcinoma	9 (69)
Favoring adenocarcinoma	2 (15)
Chronic inflammation with fibrosis	2 (15)
**Final diagnosis, *n* (%**)	
Hilar cholangiocarcinoma	9 (69)
Recurrent IHD cholangiocarcinoma	1 (8)
Pancreatic cancer	1 (8)
HCC combined with cholangiocarcinoma	1 (8)
Gallbladder cancer	1 (8)

ERCP = endoscopic retrograde cholangiopancreatography, HCC = hepatocellular carcinoma, IHD = intrahepatic duct.

**Table 3:** Summary of the loop-tip guidewire-assisted forceps biopsy results for the enrolled patients.

Patient No.	Sex	Age (yrs)	Location	Time (sec)	Histologic results	Final diagnosis
1	M	59	CHD	273	Favoring adenocarcinoma	Hilar cholangiocarcinoma
2	F	50	CHD	99	Adenocarcinoma	Hilar cholangiocarcinoma
3	M	52	Right IHD	139	Adenocarcinoma	HCC combined cholangiocarcinoma
4	M	84	CHD	214	Adenocarcinoma	Hilar cholangiocarcinoma
5	M	64	CHD	144	Adenocarcinoma	Hilar cholangiocarcinoma
6	M	69	CHD	188	Adenocarcinoma	Hilar cholangiocarcinoma
7	F	61	Left IHD	412	Chronic inflammation	Pancreatic cancer with liver metastasis
8	M	78	CHD	199	Adenocarcinoma	Hilar cholangiocarcinoma
9	M	83	CHD	158	Adenocarcinoma	Hilar cholangiocarcinoma
10	M	79	CHD	134	Adenocarcinoma	Hilar cholangiocarcinoma
11	F	69	CHD	133	Favoring Adenocarcinoma	Hilar cholangiocarcinoma
12	F	82	CHD	169	Adenocarcinoma	GB cancer
13	M	56	Left IHD	247	Chronic inflammation	Recurred intrahepatic cholangiocarcinoma

CHD = common hepatic duct, GB = gallbladde, HCC = hepatocellular carcinoma, IHD = intrahepatic duct.

## 4. Discussion

Endoscopic access to malignant tumors of the bile duct has become possible due to advancements in ERCP techniques.^[9]^ The most effective method to enhance diagnostic yield is by performing an additional endoscopic forceps biopsy to obtain a deep layer of the bile duct epithelium.^[10]^ The diagnostic sensitivity of forceps biopsy sampling and brush cytology for detecting cholangiocarcinoma in bile duct stenosis is 44% to 89%, while that for detecting pancreatic cancer is 30% to 65%.^[11]^ Because of this low sensitivity, other methods are sometimes used instead of biopsy by ERCP. However, although it is not a common case, clinically it is necessary to confirm the pathology of the primary site. If the location of the bile duct stenotic lesion is difficult to access, novice endoscopists have trouble performing the biopsy procedure. To overcome this limitation, there is a method using Histoguide® (Steris Corporation, Ohio) or with the help of the SpyGlass™ DS System (Boston Scientific, Massachusetts). However, due to differences in medical environments between countries, the routine use of single operator cholangioscopy, an expensive device, is not common yet.

Many previous studies have evaluated easy and safe ways to insert biopsy forceps into target lesions located in the bile duct.^[12,13]^ However, these methods require a specially manufactured device or can only be performed by ERCP experts. Subsequently, additional methods for the simpler insertion of biopsy forceps without the need for a special device have been developed.^[14,15]^ However, while these methods are thought to be very simple and safe during the biopsy forceps insertion process, guidewire displacement may occur during biopsy forceps retrieval because the tip of the biopsy forceps moves with the pre-installed guidewire in the bile duct.

Therefore, our research team decided to develop a method free from the issues noted by previous studies while maintaining the advantages. We therefore attempted to devise a method to insert the biopsy forceps easily and comfortably, without the need for a special device. First, we judged that assistance from a pre-installed guidewire for the biopsy forceps approach, as previously introduced by other studies, was the most effective way for forceps to easily be passed through the papilla and inserted into biliary stricture lesions, and this approach did not require any specially designed devices.^[16]^ Further, we decided to try using the loop-tip wire to reduce the risk of pre-installed guidewire displacement. During the process of forceps retrieval, it was assumed that pre-installed guidewire displacement would not occur if the biopsy forceps could be separated from the pre-installed guidewire. We performed a simple pretest with a transparent tube set as a virtual bile duct, and, based on the results of the pretest, we determined that it could be effective and safe (Supplemental Video, http://links.lww.com/MD/H399).

Despite these advantages, the loop-tip guidewire technique has obvious weaknesses. The first weakness is that a special instrument, the loop-tip guidewire, is required. The second is that although is very easy to pass forceps through the papilla using the loop-tip guidewire, difficulties remain when the lesion is in the left IHD. When the CHD and left IHD are at an acute angle, it is often impossible to access the left IHD using biopsy forceps alone. Since the biopsy forceps do not bend well, it is still not easy to access the left IHD, even with the help of the loop-tip guidewire; however, attempting to access it was easier using the loop-tip guidewire. Additionally, in the case of metastasis or invasion of other primary cancers, it is inevitable that the sensitivity of intra-ductal biopsy during ERCP is not high. This is not only a problem of loop-wire biopsy, but a limitation of ERCP biopsy. Therefore, patients 7 and 13 in Table 3 eventually had to undergo US-guided liver biopsy and 2nd look ERCP, respectively (Patient 7; liver metastasis became prominent, Patient 13; malfunction of stent inserted at 1st ERCP).

This study was not a randomized trial; therefore, another limitation is selection bias resulting from the enrollment of cases based on a novice endoscopist not successfully approaching the bile duct stricture lesion using biopsy forceps within 5 minutes. Therefore, we were able to attempt this new bile duct approach method using biopsy forceps only in a small number of cases. The aim of the study was to introduce a novel method, and a large-scale future study is still needed to confirm its clinical significance.

Although the number of subjects in this study was small, the sensitivity was clearly improved. However, while our sensitivity results were superior to those published in a previous review study,^[3]^ we do not intend to claim superior sensitivity. Since the sample size was so small, it was difficult to obtain statistical significance. However, we intended to share this method since it is a more convenient biopsy forceps approach into the bile duct that involves using a commercially available device in a manner different from its original purpose.

In summary, this biopsy forceps approach using a loop-tip guidewire is an easy and safe method to access stricture lesions in the bile duct along a pre-installed guidewire. This approach allows for the biopsy forceps to be separated from the pre-installed guidewire so that it can be moved more freely in the bile duct compared to that in other approaches. We recommend this method be used to facilitate access to bile duct stricture lesions that are difficult to access using the conventional method.

## Acknowledgment

This study was supported by Korea University and the National Research Foundation of Korea (NRF) grant funded by the Korea government (MSIT) (No 2021R1H1A2094560).

## Author contributions

**Conceptualization:** Jae Min Lee.

**Data curation:** Kang Won Lee.

**Formal analysis:** Kang Won Lee.

**Investigation:** Hyuk Soon Choi, Hong Sik Lee.

**Project administration:** Jae Min Lee.

**Resources:** Hoon Jai Chun.

**Software:** Eun Sun Kim.

**Supervision:** Bora Keum, Hong Sik Lee.

**Validation:** Yoon Tae Jeen.

**Writing – original draft:** Kang Won Lee.

**Writing – review & editing:** Kang Won Lee.

## Supplementary Material


